# 3D objects reconstruction from frontal images: an example with guitars

**DOI:** 10.1007/s00371-022-02669-x

**Published:** 2022-09-15

**Authors:** Alejandro Beacco, Jaime Gallego, Mel Slater

**Affiliations:** EventLab, Universitat de Barcelona, Barcelona, Spain

**Keywords:** 3D guitar reconstruction, Guitar segmentation, 3D objects reconstruction

## Abstract

This work deals with the automatic 3D reconstruction of objects from frontal RGB images. This aims at a better understanding of the reconstruction of 3D objects from RGB images and their use in immersive virtual environments. We propose a complete workflow that can be easily adapted to almost any other family of rigid objects. To explain and validate our method, we focus on guitars. First, we detect and segment the guitars present in the image using semantic segmentation methods based on convolutional neural networks. In a second step, we perform the final 3D reconstruction of the guitar by warping the rendered depth maps of a fitted 3D template in 2D image space to match the input silhouette. We validated our method by obtaining guitar reconstructions from real input images and renders of all guitar models available in the ShapeNet database. Numerical results for different object families were obtained by computing standard mesh evaluation metrics such as Intersection over Union, Chamfer Distance, and the F-score. The results of this study show that our method can automatically generate high-quality 3D object reconstructions from frontal images using various segmentation and 3D reconstruction techniques.

## Introduction

Methods for reconstructing 3D objects from 2D images and videos have undergone remarkable improvements in recent years. In general, these proposals use specific databases for each object type, although there is a trend toward developing general methods that compute 3D reconstruction for each object type [1, 2].

We are interested in 3D reconstruction of objects that appear in images, so that the resulting models can help improve realism in virtual recreation of events in which these images appear, such as a rock concert [3]. Our goal is to develop a method for reconstructing objects that people can interact with in scenes and enabling their subsequent use in the 3D world.

In this paper, we focus specifically on guitars as the main example. Their recognition and reconstruction are central to complementing the 3D reconstruction of guitarists in concert sequences. Research in the musical context we discuss is still quite young [3, 4], and efficient modeling of the objects is crucial to enable realistic reconstructions and interactions with 3D avatars.

We limit our reconstruction to frontal views, as this is the only angle from which we can obtain and infer sufficient information about the actual shape and appearance of the guitar. The obtained 3D reconstruction will not perfectly match the original guitar in all its dimensions and finer details, but our resulting model resembles the original with such high accuracy that it can be used in 3D virtual applications as an identifiable replica of the original object. As shown in this work, the proposed method can be adapted to almost any other object family by replicating the process with specific object data.

## Related work

3D reconstruction of objects from frontal images is an important task that mainly involves two areas of image processing: Object segmentation and 3D reconstruction.

### 2D object segmentation

Segmentation of objects requires preservation of resolution-related information and extraction of scale-related features. Among these variants, atrous convolution [5], encoder–decoder models [6, 7] and depth-wise convolution [8] have emerged and improved the performance of early CNN architectures. Depending on the task, new strategies follow hybrid approaches to exploit the best features of each method. In [9], semantic segmentation methods based on encoder–decoder networks such as DenseNet [10], DeeplabV3+ [5] and PGN [11] are used to segment guitars from images. Encoder–decoder networks usually consist of two phases: First, the feature maps are reduced to capture the semantic information; then, the spatial information is recovered by upsampling techniques. This approach has proven successful in segmentation [6, 7, 12, 13]. The Xception module, which modifies Inception V3 to improve performance on large data sets, is now used as the main backbone in server environments [5]. In encoder architectures, low-resolution features are separated from higher-resolution ones and recovered using the decoder. According to another approach, high-resolution representations should be maintained throughout the process by using a parallel network that connects the parts of the process and helps to reconstruct these features at the end. Recent work on high-resolution networks (HRNet) [14, 15] has shown very good performance.

### 3D object reconstruction

RGB image-based 3D reconstruction methods using convolutional neural networks (CNNs) have attracted increasing interest and shown impressive performance. Han et al. provided an overview of these methods in [16]. Hepperle et al. examined the quality of 3D reconstruction quality to enhance the experience in VR applications in [17].

The development of deep learning techniques, and in particular, the increasing availability of large training data sets, has led to a new generation of methods capable of recovering the 3D geometry and structure of objects from one or more RGB images without the complex process of camera calibration.

#### Volumetric representation techniques

Volumetric representations partition the space around a 3D object into a 3D grid and allow existing deep learning architectures developed for 2D image analysis, such as encoder–decoders, to be used for 3D processes.

Some methods deal with 3D volume reconstruction in the form of a voxelized occupancy grid, for example, in [18–20]. In general, voxelized representations lack accuracy due to the high memory requirements for reconstructing large voxel grids, and other methods attempt to solve the 3D object reconstruction problem using meshes. This is the case with Mesh-RCNN, the work presented by GKioxary et al. in [2]. In this work, the authors propose to predict coarse voxel representations that are transformed into meshes. In [1], Wen et al. developed a method inspired by traditional multiview geometry methods. Mescheder et al. proposed occupancy networks in [21], introducing a new representation for 3D geometry. Unlike existing representations, occupancy networks are not constrained by the discretization of 3D space.

Methods based on volumetric representation are computationally intensive. They cannot provide sufficient quality for use in 3D virtual productions due to memory requirements and limited grid size. In addition, they cannot correctly handle textures that need to be added in later steps.

#### 3D Differentiable rendering

Differentiable rendering has been used to solve various 3D-related problems, such as reconstructing 3D objects from a single 2D image. These techniques allow the gradients of the rendering process of 3D objects to be computed and propagated through images. Kato et al. published a survey of differentiable rendering methods in [22].

Jiang et al. presented a differentiable rendering approach for rendering 3D geometry called SDFDiff in [23], which provides 3D models without texture information. Sitzmann et al. proposed in [24] to learn the rendering process from data using neural rendering. Although differentiable rendering methods provide promising results, the quality of the reconstruction and the associated textures currently do not provide the quality and realism required to use the reconstructed 3D objects in realistic 3D scenarios.

#### 3D reconstruction using template deformation

These methods use specific 3D models adapted to the object image to be reconstructed. For example, in [25], the authors construct an embedding space and use a CNN to determine the models that are most similar to the input object. In [26], the authors used deformable 3D models matched to images based on pose and silhouette estimates.

Since this method performs an adjustment in 3D space, which is difficult to perform, the results obtained lack precision. The shapes of the resulting models are not accurate and texture cannot be applied to the resulting mesh. The authors of [27, 28] use CAD models to estimate the 3D shape of an object using a set of landmarks. Zou et al. focused in [29] on finding the most similar 3D shapes from a 3D shape space for input image queries.

The problem with these methods is that the deformation is performed in 3D space and the results do not match the exact shape of the object.

Pan et al. in [30] proposed to reconstruct the 3D mesh of an object by deforming a sphere, while Wang et al. in [31] used a similar approach with an ellipsoid template. Both methods achieve correct results in the final shape representation, but they do not deal with realistic texture to complete the final model of the objects. In general, methods based on template deformation can reconstruct 3D objects that correctly match the input images, but they are not able to reconstruct a complete texture because it is difficult to establish mesh-texture correspondences to correctly match the realistic texture of the image to the created 3D model.

We have developed a system corresponding to the last group of techniques, based on the deformation of templates that allow a 3D reconstruction that better matches the actual shape and texture of the objects from the 2D images, to obtain a photorealistic 3D reconstruction. We work with templates for each category of objects, which allow us to assign and use inherent features of the objects to correctly fill in the sides and dimensions hidden in the 2D images. In addition, the animations and higher-level properties associated with the template are preserved in the 3D reconstruction and can be used later in a 3D model animation system.

## Method

**Fig. 1:**
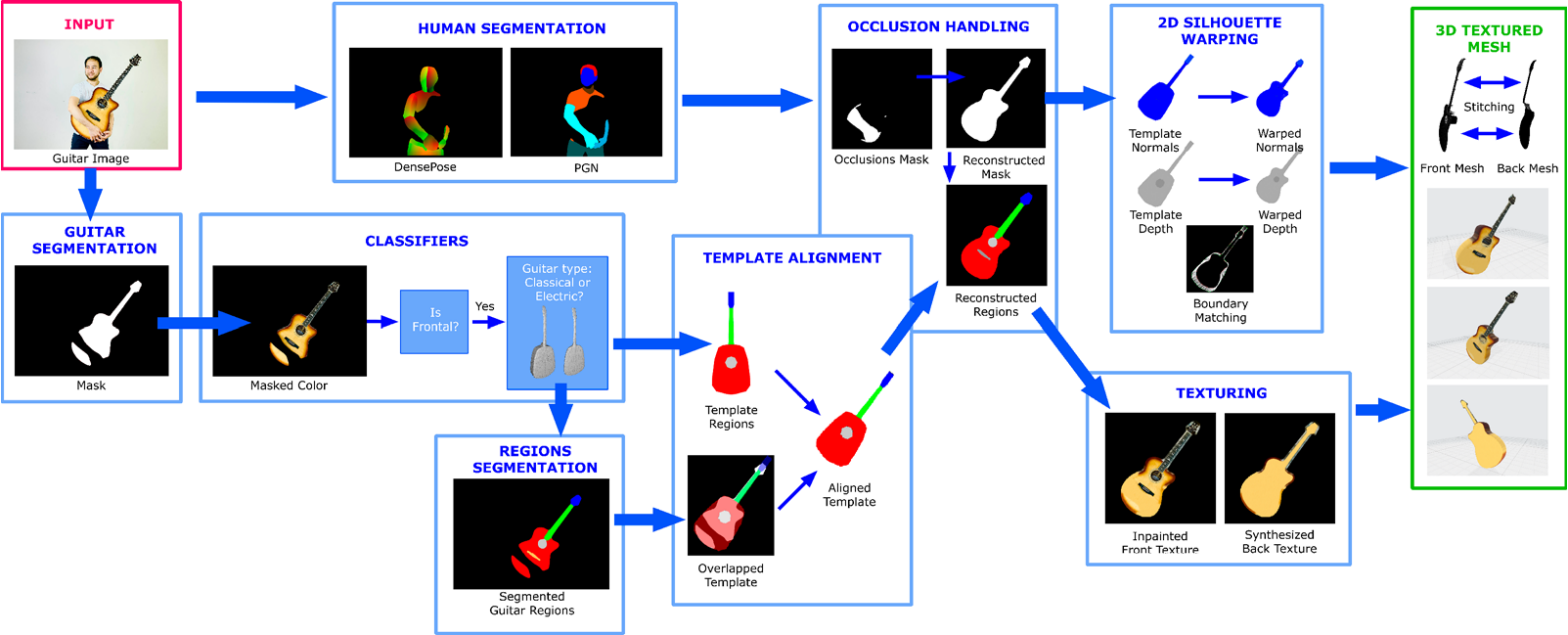
Workflow of the proposed system

Our system inputs a monocular 2D RGB image and outputs a fully textured 3D mesh of the object. Figure 1 illustrates this workflow using a guitar as an example.

First, the guitar that appears in the image is detected and segmented. Second, the segmented guitar is used to decide whether the viewpoint is frontal or not, and if so, what kind of guitar we are dealing with: a classical Spanish guitar or an electric guitar. We have chosen to divide the guitars into two families because there are important morphological differences between them. In a third step, we segment the inner regions of the guitar, which helps us to align the silhouette of the corresponding guitar template in 2D. To detect possible occlusions, we segment people in the image using two methods [11, 32] to compute an occlusion mask. Using the aligned template silhouette, we reconstruct the occluded parts of the mask and also reconstruct the segmentation of the regions.

Finally, 3D reconstruction is performed by warping the front and back depth and normal renders of the aligned template in 2D space to fit the reconstructed silhouette to the input. The resulting mesh is obtained by back-projecting the resulting depth maps, while the original image is projected as a frontal texture (with inpainted occluded regions) and the back texture is synthesized from it.

### Guitar segmentation and classification

We defined a set of segmentation and classification methods to extract the information of the guitar region appearing in the image and to verify that the guitar to be reconstructed meets the minimum processing requirements. We follow a framework of weak classifiers that can be combined sequentially to simplify the creation of the databases and their generalization to various other objects.

The proposed method starts with the segmentation of the guitar from the image, and then, a chain of classifiers and segmentation methods is applied to this first segmentation: We classify the segmented guitar into frontal/non-frontal classes to check whether the guitar is frontal to the camera. If the classification reveals that the guitar is frontal, the process continues with a second classifier that detects whether the guitar is electric or classical. This determines the type of template needed to correctly fit and reconstruct the guitar model. Finally, another segmentation is performed to extract the detected regions of the classical/electric guitar. This segmentation is used to align the 3D template with the orientation of the guitar and improve edge fitting during 3D reconstruction.

#### Guitar/non-guitar segmentation

To obtain a correct 3D reconstruction, an accurate segmentation of the guitar is required. We use the database and segmentation presented in [9] with 2, 200 RGB images of guitars (11,000 images after enhancement) to train and test the selected network. We randomly select 80% of the original data for training and the remaining 20% for testing. This database is also used for the classification methods explained in the following sections.

To obtain the best segmentation, we performed a full evaluation for three of the best CNNs for segmentation: Deeplabv3+ [5], HRNet [10], and PGN [11], where each CNN was trained from scratch with 40,000 iterations.

**Table 1:** Comparison on evaluation sets applying 40K training iterations

Network	Crop	Batch	mIoU (%)
DeeplabV3+	513	8	88.47
HRNet-C1	$960\times 720$	4	95.31
SCI-PGN	512	4	96.61

**Fig. 2:**
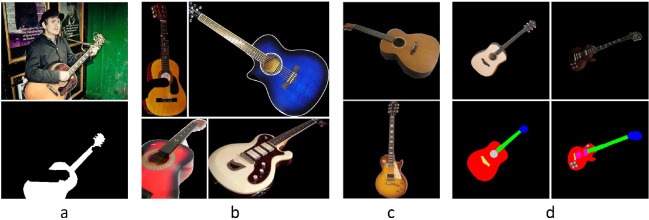
Examples of classification and segmentation. From left to right: **a** segmentation of guitar with [9]. **b** Frontal (top) and non-frontal (bottom) guitars. **c** Classical (top) and electric (bottom) guitars. **d** Labeling of the segmentation of the regions

The performance of all networks with 40K iterations is shown in Table 1. As we can see, DeepLabv3+, HRNet and PGN achieved Mean Intersection Over Union (mIoU) of 88.47%, 95.31% and 96.61%, respectively. Figure 2a shows an example of the guitar segmentation achieved.

Therefore, in our implementation, the PGN network is chosen to obtain a high-quality object segmentation. In Fig. 11, second column, we can see examples of guitar segmentation results obtained with this network.

#### Frontal/non-frontal guitar classification

To detect whether the guitar segmented in the previous step is frontal enough to be processed by our method, we developed a frontal/non-frontal classifier based on CNNs.

We use the guitar segmentation obtained in the previous step, cut into a square block with a black background, as input to our classifier to determine whether the guitar image is frontal or non-frontal. We use a CNN reference model that has shown correct classification results based on its appearance: ResNet50, a 50-layer residual network with correct performance on classification tasks [33].

Our database consisted of 2,397 images (some images in the dataset presented in [9] contained more than one guitar), of which 989 were frontal and 1,408 were non-frontal, and was augmented obtaining 123,625 frontal and 151,000 non-frontal images. We prepared the images with a masking effect so that the guitars had a black background. We randomly selected 80% of the data for training and the remaining 20% for testing. The data were expanded by a combination of rotations every $15^{\circ }$ (15,30,45,...,245) and horizontal flipping, and by four color modifications using Reinhard’s color normalization [34]. The final number of samples per class after data augmentation was 123,625 frontal images and 151,000 non-frontal images.

Figure 2b shows an example of the images used in this classification process.

We adapted the database to the ResNet50 model and trained it on a GPU NVIDIA Titan X with 24 GB, with 6 epochs, a batch size of 16, stochastic gradient descent optimizer, and a learning rate of $10^{-4}$. With this configuration, this CNN achieved a classification accuracy of 99.4%.

#### Classical/electric guitar classification

In our proposal, we use a different 3D template for classical and electric guitars to better fit the system to the actual shape of the instrument. Thus, we need to determine what type of guitar it is so that we can apply the correct template. From the 989 frontal guitars extracted in Sect. 3.1.2, we obtained 470 and 519 classical and electric guitars, respectively, of which 80% were randomly selected for training and the remaining 20% for testing. We then augmented them obtaining 58,750 classical and 64,875 electric guitars. Figure 2c shows sample images from this dataset. ResNet50 was trained with the same configuration as in Sect. 3.1.3, obtaining 98.3% accuracy.

#### Regions segmentation

This step is used to match the 3D template with the parts of the object, so that each region can be correctly located and placed when reconstructing the final 3D model. For each guitar type, we define different regions:

- Classical guitar, five regions: Head, Neck, Body, Bridge and Hole.
- Electric guitar, six regions: Head, Neck, Body, Bridge, Pickups and Controls.

Figure 2d shows a graphical representation of these regions.

To identify these regions in the segmented guitar, we use the PGN model [11] to implement CNN segmentation, as this model has shown better performance in guitar segmentation.

Since we were working with controlled images with low variability, frontal guitars on a black background, two synthetic databases were created to automatically annotate the guitars. We used twenty 3D guitar models, manually labeled their textures, and rendered the 3D models. To create variability in the patterns, we performed 1000 random rotations to obtain a frontal perspective: $0<x<15$, $0<y<360$, $0<z<15$. We also rendered the guitars lit from random light directions and unlit with only diffuse color. For each classical and electric database, 200,000 images were created with the corresponding labels. After training the PGN model, we achieved an accuracy of 89.3% and 92.3% for the electric and classical classes, respectively.

### 3D reconstruction

Based on [35, 36], which deals with 3D reconstruction of people, our method computes 3D reconstruction of rigid objects, in our case guitars, appearing in RGB images. The following sections describe all the steps performed to obtain our final 3D mesh reconstruction using as input the guitar type, the segmented guitar, its mask and its segmented inner regions.

#### Aligning template renders

**Fig. 3:**
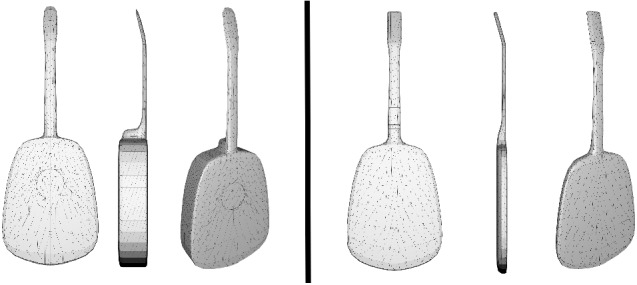
Templates of classical (left) and electric (right) guitars

By knowing the type of guitar (classical or electric), we choose the template to use. These templates are specially designed to facilitate the reconstruction process: They contain smooth surfaces on the sides, without unnecessary cavities, mainly flat surfaces on the front and back, without small details like the strings or buttons. The main differences between them are the thickness and the sound hole. Figure 3 shows the design of the two templates.

Using two templates to model different categories allows us to include differential aspects between classes that improve the realism of the 3D reconstruction for each guitar type and allows us to preserve specific features, animations, and blend shapes. This also helps to adapt the system to each generic object by simply using the right template. These templates can be easily created using modeling techniques or even downloaded from existing repositories. //

Depth and normal map renders Since we are only considering frontal views, we can use the same depth and normal map renderings for front and back for all reconstructions of a guitar type. Thus, these are rendered once and stored along with the camera view and projection matrices. It is important to note that the front and back renders must have the same silhouette: We want to apply the same deformation (warping) to both later. This can only be achieved by either using an orthogonal camera or performing an inverted rendering of the back faces (using front-face culling, disabling back-face culling and inverting the depth test).

Alignment The pre-rendered depth and normal maps for the front and back of our template are aligned to the input mask. We scale and rotate them to maximize the overlap between the two masks and apply a rigid registration method based on mutual information analysis [37, 38]. Since our templates are symmetric about the YZ plane, we do not need to check for a mirror transformation.

#### Boundary matching

The template is not a complete reconstruction of the model we are dealing with, but a rough approximation of the shape of one. Therefore, after aligning the template and the input silhouettes, we still need to find a boundary matching and perform silhouette warping in order to obtain any shape from the entire spectrum of possible shapes.

**Fig. 4:**
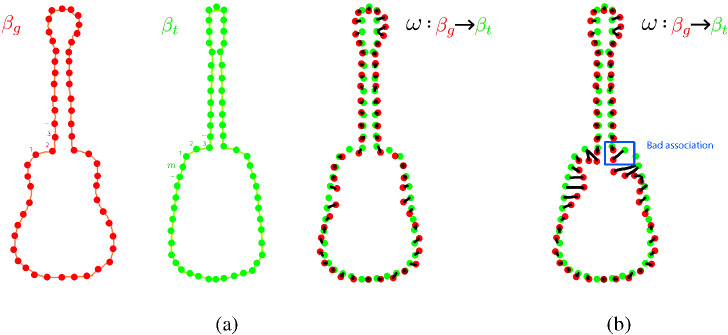
Boundary matching between the silhouettes of our guitar template (green) and an input guitar (red). Each point represents a pixel of the boundary. **a** Boundary matching associations; **b** example of a bad association, where a pixel of the input guitar’s neck is associated with a pixel of the template guitar’s body

We need to find a boundary matching $\omega$ between the silhouettes of our guitar template and the input guitar (see Fig. 4a). Given the contour of the segmented guitar $\beta _g$, the pixels $p_g \in \beta _g$ belonging to this contour, the contour of our template $\beta _t$ and the pixels $p_t \in \beta _t$ belonging to this contour, we want to warp $\beta _t$ to its counterpart $\beta _g$ to match the template to the real shape of the object. We are looking for a mapping $\omega$ that defines the correspondence between the pixels belonging to $\beta _g$ and $\beta _{t,\omega }$ by minimizing the distance between all the associated pixels of the contour of the template and the real contour of the segmented guitar:

1 $$\begin{aligned} \mathrm{arg min}_{\omega [0],...,\omega [m-1]} \sum _{i=0}^{m-1} \Vert (p_{g,i},p_{t,\omega [i]})\Vert _2 +\sigma (\omega [i],\omega [i+1]), \end{aligned}$$

where *m* is the number of pixels of the contour $\beta _t$ and

2 $$\begin{aligned} \sigma (\omega [i],\omega [i+1]) = \left\{ \begin{array}{l} 1, \quad \,\,\text {if}\,\,\, 0\le \omega [i+1]-\omega [i] \le k \\ \infty , \quad \text {otherwise} \end{array}\right. \end{aligned}$$

Therefore, $\sigma (\omega [i],\omega [i+1])$ penalizes jumps between associations larger than *k* pixels. In our implementation, $k = 128$ leads to correct results, but this is closely related to the working resolution we use (at most 350 $\times$ 350).

Depending on the value of *k* and the shape of the guitar, bad associations may occur, for example, when a pixel of the guitar’s input neck is matched to the guitar template’s body (see Fig. 4b).

To solve this problem, we use the computed segmented regions. Boundary matching $\omega _R$ is then computed for the individual masks of each region *R* (with a smaller constraint $K = 32$), and the resulting mappings can be combined. Each pixel $p_g$ of the original silhouette $\beta _g$ also belongs to the boundary $\beta _{g,R}$ of at least one segmented region *R*, but a mapped pixel $p_{t,R} \in \beta _{t,\omega }$ may or may not belong to the original silhouette of the template $\beta _t$. We therefore keep those that belong to $\beta _t$, obtaining an initial mapping $\omega _\mathrm{init}$ whose gaps can be easily filled. Let *u* and *v* be two indices of $\beta _g$ that have a mapping $\omega _\mathrm{init}[u]$ and $\omega _\mathrm{init}[v]$ belonging to $\beta _t$, e.g., $u < v$ and $s = v - u$. We find a mapping point in $\beta _t$ proportional to all indices between *u* and *v* by decomposing the segment of $\beta _t$ between $\omega [u]$ and $\omega [v]$ into *s* parts.

#### Occlusions

To solve the possible occlusions that musicians can create on guitars, we need to find an occlusion mask that indicates which parts of the guitar are occluded, but we also need to reconstruct the occluded parts of the boundary to get a reconstructed guitar mask. Finally, the map of the segmented regions should also be extended to cover the reconstructed mask.

Occlusion mask We use PGN [11] to segment the human silhouettes in the image and combine it with the output of Densepose [32] to determine which parts of the human silhouette correspond to arms and hands. Note that, we only consider occlusions by these parts of the human body, although the rest of the method could be applied to other possible occlusions by other body parts or objects (if we detect them).

**Fig. 5:**
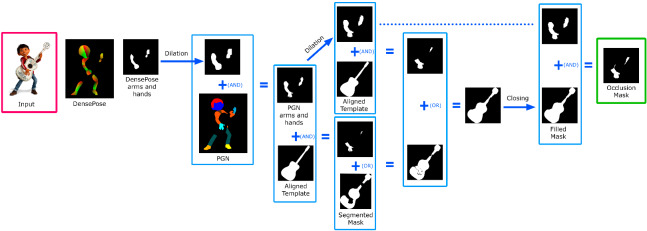
Procedure for calculating the occlusion mask

Figure 5 illustrates the process of calculating the occlusion mask. We compute the DensePose [32] segmentation of the human and select only the arms and hands from it. After dilation, this mask is combined with the more accurate PGN human segmentation [11]. The resulting mask corresponds to the parts of the arms and hands of the PGN segmentation. This is combined on one side with the silhouette of the aligned template and then with the segmented input mask of the guitar. On the other side, the arms and hands of the PGN mask are dilated and combined with the aligned template. The two resulting masks are combined again and closed to obtain a filled output mask. A final operation with the dilated arms and hands results in a first occlusion mask.

**Fig. 6:**
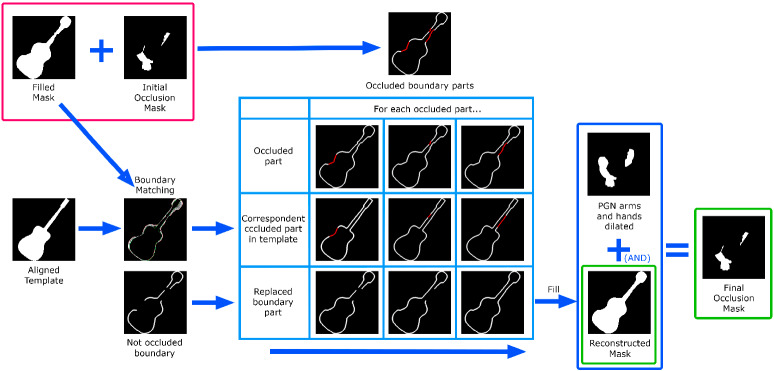
The initial occlusion mask allows us to find the occluded parts of the boundary of the previously filled mask

Silhouette reconstruction To reconstruct the occluded parts of the boundary, we find the corresponding boundary parts of the rendered guitar template (using the boundary matching algorithm explained earlier in Sect. 3.2.2). We replace each of the occluded boundary parts with the corresponding part of the template using a similarity transformation defined by the end points of each part. After reconstructing the mask of the guitar, we recombine it with the human segmentation to obtain a final occlusion mask. Figure 6 illustrates this process.

**Fig. 7:**
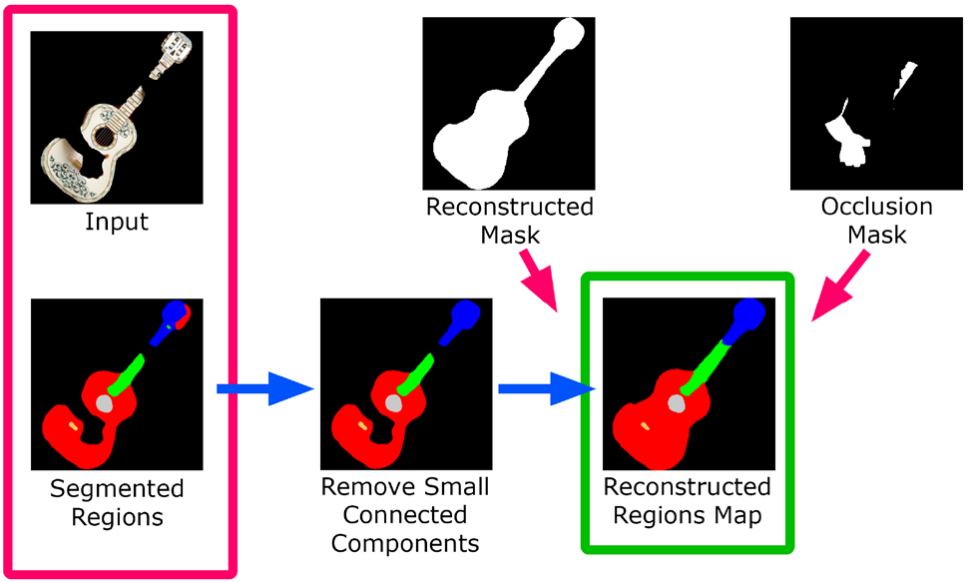
The map of segmented regions is reconstructed using two graph cuts regularizations [39] to fill the smallest connected components of each region and the occluded parts of the guitar mask

Regions map reconstruction After obtaining the reconstructed guitar mask and the final occlusion mask, we also need to reconstruct the map of the segmented regions, so that their silhouette matches that of the reconstructed guitar mask. First, for each differently labeled region, we keep only the largest connected component available in the current map of regions. For the smallest connected components, we use the $\alpha$-expansion [39] (a distance-based graph cuts regularization) to fill them, as in [35, 36]. Finally, we fill the occluded parts from the occlusion mask in the same way and obtain the final map of the reconstructed regions (see Fig. 7).

#### Silhouette warping

Following [35], the renders of the template are warped in 2D space to fit the silhouette of the input mask. This 2D warping is applied to both the front and back renders so that the resulting warped maps still have the same silhouette.

The current warping only considers the global silhouette of the guitar, but this does not ensure that the warped inner elements, such as the sound hole, are correctly overlapped with the corresponding element in the input color image (which is later projected as a texture). With the map of segmented regions, we can improve our warping with Moving Least Squares [40] by using the boundary pixels of each region and their matching pixels from the template (which are in turn computed using the boundary matching algorithm from Sect. 3.2.2) as pivots.

#### Meshing

**Fig. 8:**
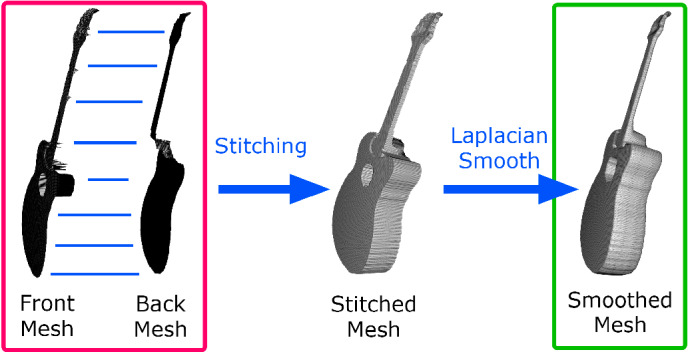
Stitching front and back meshes

We unproject each pixel of the warped depth maps to obtain the corresponding 3D vertex. Since warping the 2D silhouette can change the X and Y dimensions (making the silhouette larger or smaller in 2D), we scale the Z dimension accordingly to maintain the proportions of the guitar in all dimensions.

We create two triangles for each square of 4 pixels and get two meshes: one frontal and one posterior, which we stitch over the silhouette (see Fig. 8).

Finally, the entire mesh is smoothed using Laplacian smoothing.

#### Texture

**Fig. 9:**
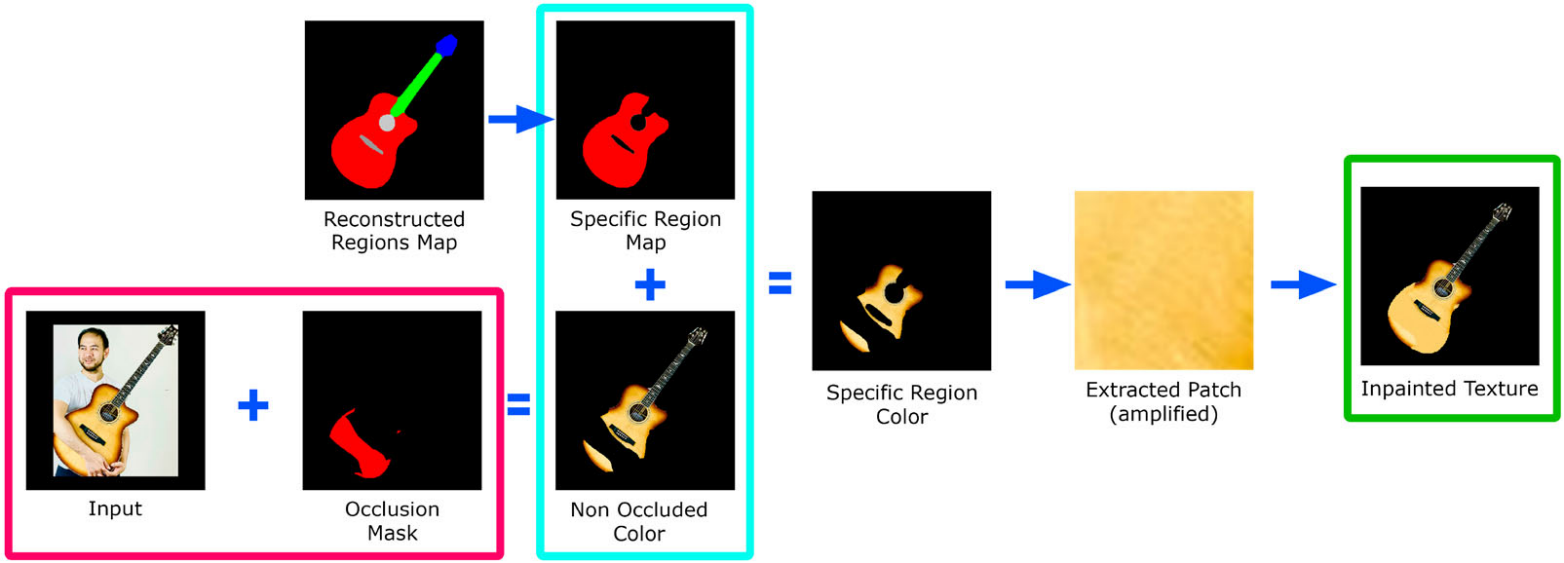
Front texture inpainting

The texture of the model can be obtained by directly projecting the texture of the masked guitar onto the front mesh. Therefore, the quality of the texture of the 3D model and its details are preserved from the original image. There are several aspects to consider in this process. First, we need to inpaint the input color image with the occlusion mask and the segmented regions in an occlusion. To do this, we fill each region of the occlusion mask by taking the largest possible patch from the unoccluded parts of the same region in the original color image. With such a patch, we synthesize a texture that covers the corresponding region of the occluded mask (using [41]), dilate it and paste it smoothly into the original occluded image. In this way, for example, guitar body is inpainted using only patches of the body. Figure 9 shows an example of this process.

We limit our texture synthesis approach to regions where we can find a sufficiently large patch (between $60\times 60$ and $100\times 100$ pixels), and otherwise use Exemplar Inpainting [42, 43].

**Fig. 10:**
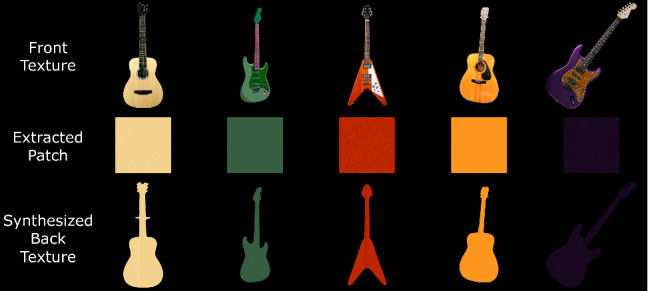
Examples of synthesized back textures along with their extracted patches

The resulting inpainted texture is then projected onto the front mesh as a color texture. For the back texture, we use a similar strategy for inpainting the occluded parts: We use [41] to synthesize a texture that covers the entire silhouette using the largest possible patch in the guitar body region of the front texture. Thus, we assume that the back of each guitar has the same color and texture as the body. Figure 10 shows several examples of back textures.

When stitching the front and back meshes, we also ensure that the corresponding front and back boundary vertices have the same UV coordinates in the final texture. This results in faces that map to the boundary of the texture as if we were stretching those pixels.

To add additional detail and relief, we also compute a bump map from the resulting front and back textures (we compute the horizontal and vertical derivatives of the grayscale textures and multiply them by a strength factor). All these texture operations are performed at the same resolution as the original input image to preserve the maximum texture quality.

## Results and evaluation

The proposed system has been tested to evaluate its quality and numerical performance compared to other reference methods.

**Fig. 11:**
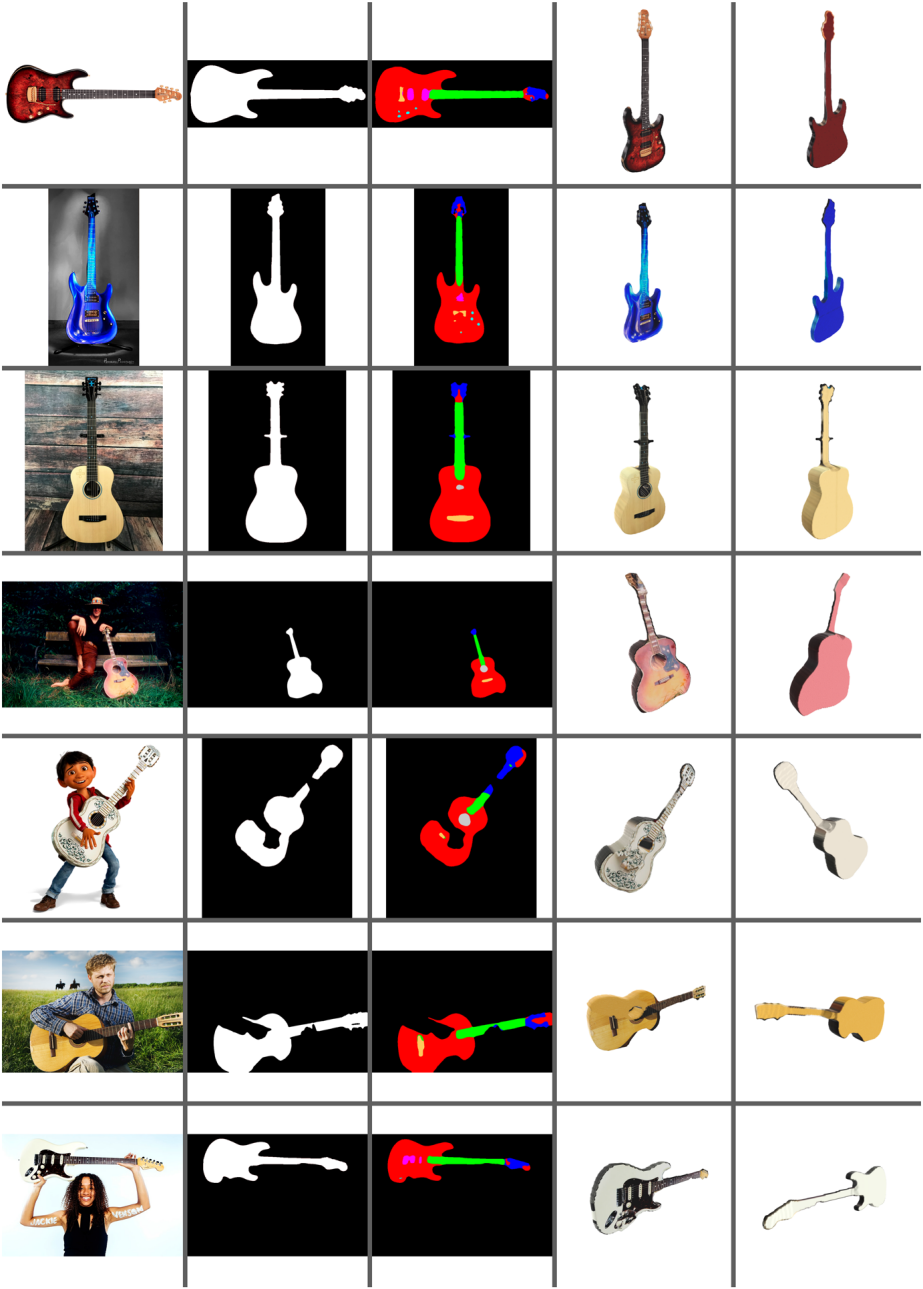
Results with Internet images. Each column shows from left to right: input image, guitar segmentation, guitar regions segmentation, and 3D reconstruction front and back views

**Fig. 12:**
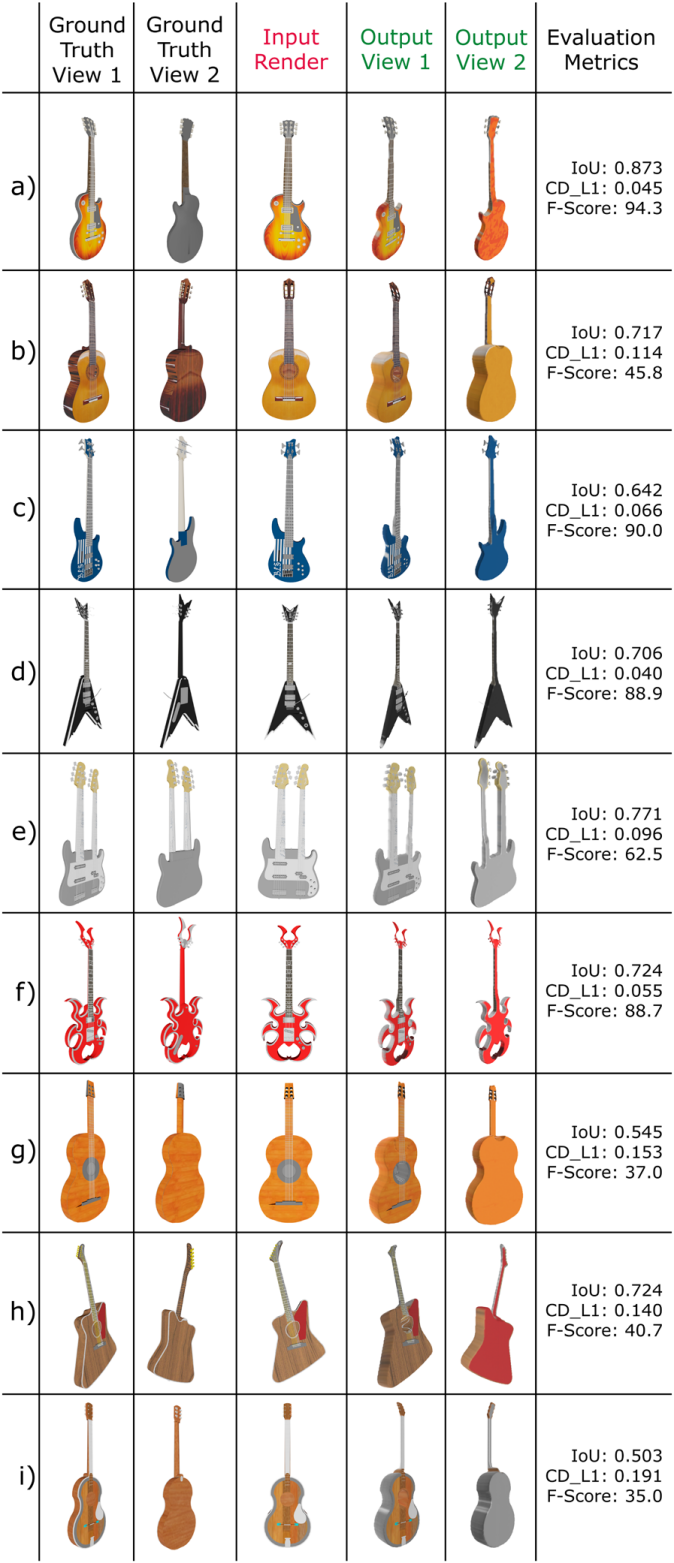
Visual and quantitative comparison between 3D ground truth guitar meshes and the obtained reconstruction using renders of these meshes as input

**Fig. 13:**
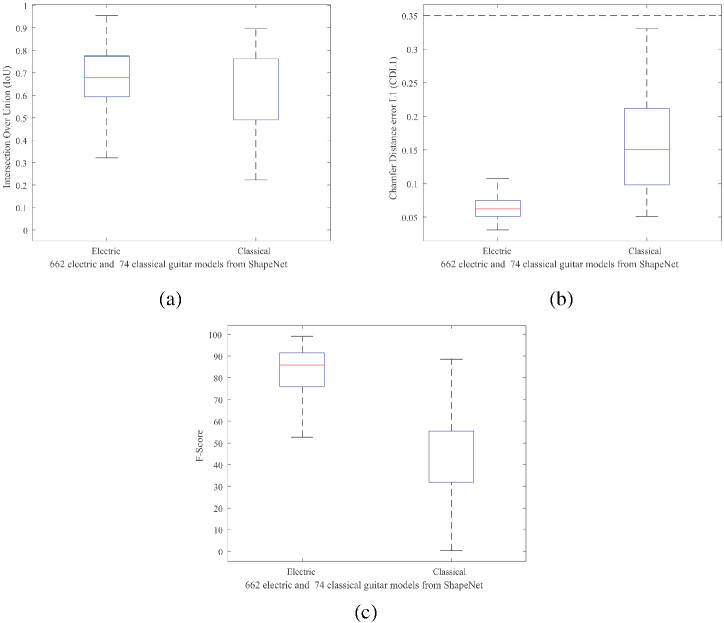
Evaluation metrics on ShapeNet guitar models

Figure 11 shows some results using some images from the Internet, while Fig. 12 compares some resulting models with the corresponding ground truth when renders of these models are used as input (those from the ShapeNet database [44]). Numerical results shown in Fig. 13 were calculated by evaluating 3D guitar models from the ShapeNet database [44]. Specifically, the folder number “03467517” from ShapeNetCore was used. We rendered 662 electric and 74 classical guitars.

We used standard metrics for comparing 3D meshes such as Intersection Over Union (IoU), Chamfer Distance (Chamfer-$L_1$) and F-Score [45, 46].

Before comparing two meshes, we center and normalize each mesh (by the main side length), voxelize it ($128^3$ voxels), and fill the inner voxels (make them solid objects). The two voxelizations are aligned using Iterative Closest Points (ICP) [47] to maximize their overlap and calculate the IoU. We then scale each original mesh by taking as its unit 1/10 times the maximum edge length of the bounding box of the current object, as described in [48]. Following [45], we uniformly take 10K points from each mesh surface and calculate the Euclidean distance between them. In this way, we can calculate the Chamfer-$L_1$, and the F-score with a threshold of 1% of the side length (i.e., 0.1). Figure 13 shows boxplots of the obtained results, while Fig. 12 shows the specific values of some reconstructions.

As for the electric guitars, the not too high IoU values show us that the resulting meshes are not extremely different from the originals, but as mentioned in [45], they also do not guarantee that the meshes are very similar. The very low Chamfer-$L_1$ values (about 0.062), obtained as the mean between completeness (mean square distance of the reconstructed model from the original) and accuracy (the opposite), prove that our reconstructions are good and very similar to the originals, although the Chamfer Distance is very sensitive to outliers. Finally, the F-score, a metric that removes outliers from the equation, shows values that support our method with very high scores (about 85%). Intuitively, the F-score value represents the percentage of points that are correctly reconstructed.

For classical guitars, the IoU values are in the middle range, which again just means that the meshes are not very different. Chamfer-$L_1$ values are higher than for electric guitars, but still quite low (around 0.15, but up to 0.35), which means that these reconstructions are also good and quite close to the originals, but with a larger distance between the surfaces. Finally, we see lower F-score values (by 40%), but this correlates with the Chamfer-$L_1$ values and our F-score threshold of 0.1. These differences arise from our reconstruction method itself, in which one dimension is completely recreated from our template. The more the thickness of the template matches that of the real guitar, the closer the two surfaces, especially the reconstructed front and back surfaces. Since electric guitars are thinner, the variations in thickness and our distance error are small. This is not the case with classical guitars, as their thickness varies more, resulting in a much larger distance between the surfaces in our evaluation.

In analyzing this result, it is important to recall once again that our system works only with a frontal image as input, and the posterior part of the reconstructed model is derived only from the template. This means that in many cases, our reconstruction does not match completely the original model. Although we try to be as accurate as possible, in many cases, this is impossible due to the lack of information. Our main goal is to obtain a plausible and valid 3D model that resembles the original guitar in the input image, a model that can later be used in any 3D production.

### Generalization

Our 3D reconstruction pipeline can be adapted to other object types by training the segmentation and classification methods and using the appropriate templates for each specific object family.

**Fig. 14:**
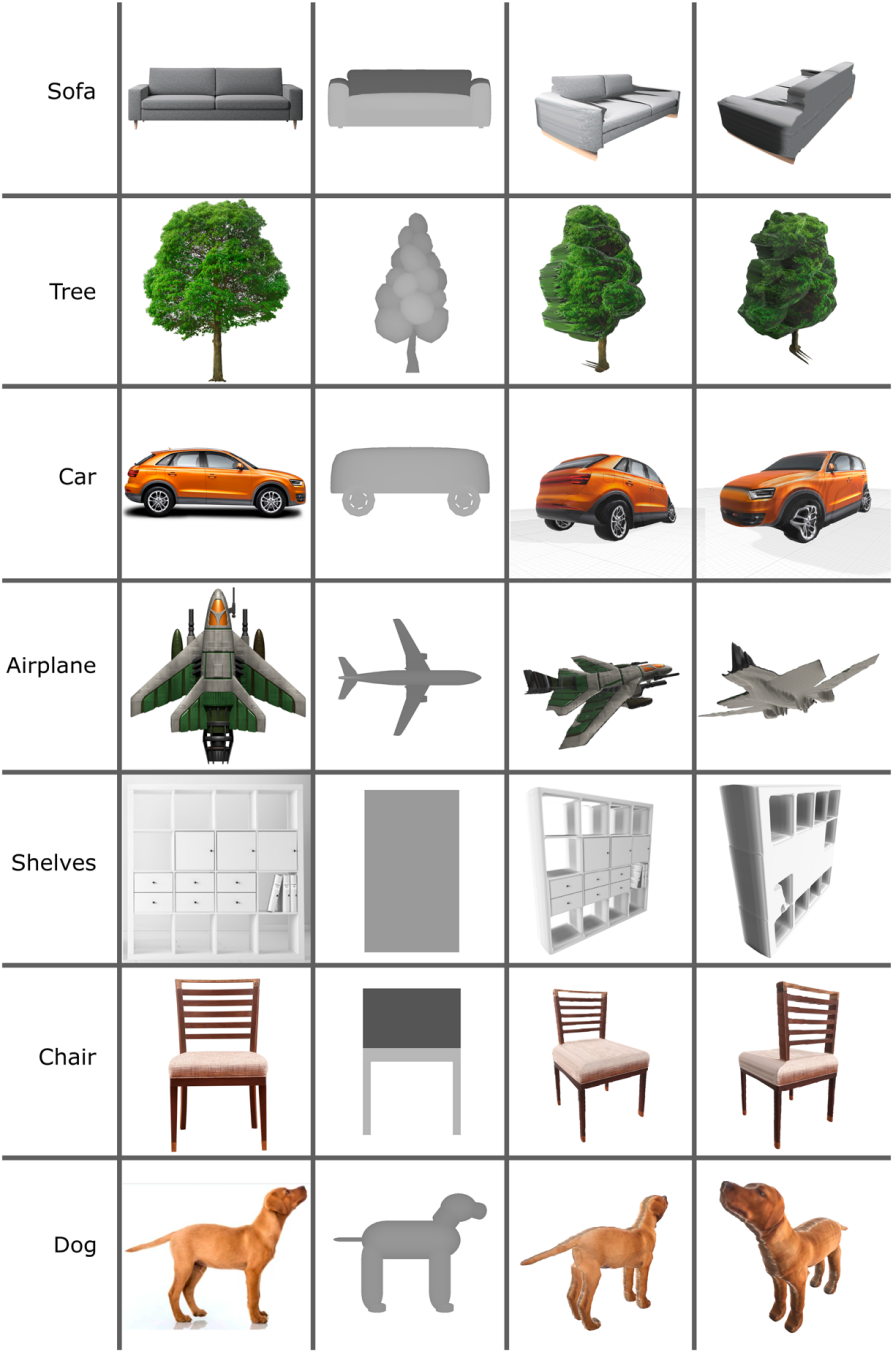
Results with images of other types of objects downloaded from the Internet. Each column contains, from left to right, the input image, the depth render of the template used and two views of our 3D reconstruction

Figure 14 shows some examples of generalization of our method assuming we have the segmentation of the object.

Different objects may require different rules or configurations for creating back textures. The “sofa,” “plane,” and “shelf” objects might use the same synthesis approach based on the largest patch like the one we used for the guitar object. But others like “tree,” “car,” “chair” and “dog” could simply use a reflection of the front texture. For some objects, it might also be beneficial to know some symmetry rules for creating the 3D mesh. For example, the car and the dog could have a symmetry rule across the XY plane, while the chair could also have that symmetry, but only on the legs. The chair and the shelf show that we also support holes in the segmented input mask. Our system can handle configurations like this and others, and we believe we can embed all these different rules for each object type.

**Fig. 15:**
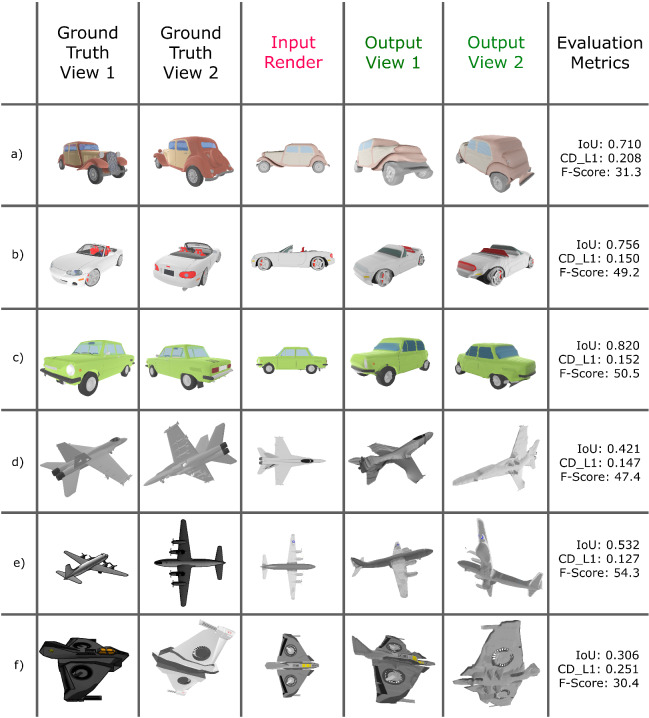
Visual and quantitative comparison between 3D ground truth meshes of cars and airplanes and the obtained reconstruction using renders of these meshes as input

Additional qualitative and quantitative results computing the IoU, Chamfer-$L_1$ and F-score values can be seen in Fig. 15, which compares our results with ShapeNet’s 3D ground truth meshes.

As can be seen in this figure, our method achieves accurate shape and realistic texture reconstruction results even when the object is very different from the base templates used for these tests (see Fig. 15a, b or f).

**Table 2:** Comparison of mean values of IoU, Chamfer-$L_1$ and F-score for reconstruction of cars and airplanes from ShapeNet using different methods. Our values are the means of 100 car and 100 airplane reconstructions

	Metric	Method						
		3D-R2N2[18]	PSGN[48]	Pix2Mesh[31]	AtlasNet[49]	OGN[50]	Matryoshka[51]	Ours
Cars	IoU	0.661	–	0.552	–	–	–	**0.699**
	Chamfer-$L_1$	0.213	0.169	0.180	**0.141**	–	–	0.195
	F-Score (%)	–	–	–	30	37	**38**	37.5
Airplanes	IoU	0.426	–	0.420	–	–	-	**0.486**
	Chamfer-$L_1$	0.227	0.137	0.187	**0.104**	–	–	0.159
	F-Score (%)	–	–	–	39	26	33	**52.5**

Best values obtained in the comparison appear in bold

Table 2 shows a comparison between our method using 100 reconstructions of two relevant object classes from ShapeNet: “car” and “airplane,” with the mean values of IoU, Chamfer-$L_1$ and F-score values obtained in these classes using the following methods: 3D-R2N2 [18], PSGN [48], Pix2Mesh [31], AtlasNet [49], OGN [50] and Matryoshka [51]. In this comparison, we see that our reconstruction achieves competitive results for cars reconstruction with 0.699 as the highest IoU score, 0.195 Chamfer-$L_1$ and 37.5% F-score.

For the airplanes reconstruction, we obtain the highest IoU score of 0.486 and the highest F-score of 52.5%, far from the 39% of the second method (AtlasNet [49]), while for Chamfer-$L_1$, we obtain a value of 0.159, which is close to the highest value of 0.104 obtained with AtlasNet [49].

Although we use a single coarse template for each class and our input view (frontal) does not include information in the depth dimension, this comparison shows that our results are comparable or even better than the state of the art. Furthermore, this comparison does not take into account that our method also adds texture to the model, which improves the realism and quality of 3D reconstructions, but this is not considered in these metrics. Classifying cars and airplanes into different subclasses associated with specific templates would improve our results and provide a more realistic reconstruction of the objects.

## Discussion

Several aspects can be discussed about our design. Concerning the 2D analysis, we use sequential classifiers instead of a single CNN trained to detect front-facing electric guitars and classical guitars. We split this process into different classifiers to simplify the creation of the databases and their generalization to various other objects, so that we follow a framework of weak classifiers that can be combined sequentially. As for the reconstruction of frontal objects, we work with frontal objects that allow a deviation of $\pm 10^{\circ }$, which can lead to some error in the reconstruction. Applying a 3D alignment of the template to support any viewpoint could be optional, but then, the captured depth maps and 2D warping could fail in certain situations. Ultimately, our method requires a viewpoint that better captures the general shape of the object with a front-back symmetry of the silhouette and whose hidden dimension has the least information loss.

Some objects are more suitable for our method than others. Objects with concavities above the hidden depth dimension or with complex internal details are not suitable because they cannot be captured by the depth maps and therefore cannot be reconstructed. In addition, the details of objects not included in the templates will not appear modeled in the final reconstruction. In the case of guitars, for example, some parts such as the strings or the controls and pickups of electric guitars were intentionally not modeled with specific structures in the template to simplify later deformations. We did not aim for such a high level of detail and relied on the quality of the input texture added to the normal mapping to simulate such details.

Failed 3D reconstructions can occur primarily when other elements of the scene affect the results. If the element to be reconstructed is occluded and the occlusion is not correctly resolved by our system, incomplete 3D reconstruction and/or texturing will occur because the occluded areas cannot be resolved. Figure 11 shows some examples in row 4, where the lower part of the guitar is not reconstructed correctly because it is occluded by the grass, and in row 7, where both hands occlude the guitar and the final reconstruction resolves these areas incompletely. If the segmentation of the guitar fails by inserting an element of the scenario into the object mask, this element can be inserted into the final 3D model. This is the case shown in Fig. 11 row 3, where the neck of the guitar is not segmented correctly and the support is inserted into the reconstruction mask.

In terms of computational cost, our implementation performs each of the steps explained in this work sequentially. Using a Windows 10 PC with an AMD Ryzen 7 3700X 8-core processor, 32 GB RAM and an Nvidia RTX 2700 GPU, our setup can generate the 3D model of a guitar from an image in about 2 min. This runtime could be optimized by parallelization and code optimization techniques.

## Conclusions

In this paper, we presented a complete system for 3D reconstruction of objects in frontal RGB images based on template deformation focusing on guitars to explain the method. It allows realistic 3D reconstruction in shape and texture and solves possible occlusions that can hide some parts of the object.

Unlike other reference methods, we work with both shape and texture and take into account occlusions present in the images. Therefore, the 3D models of our reconstructed guitars are accurate and realistic and can be used in 3D virtual reconstructions. Moreover, we have shown that our pipeline can be adapted to other objects, provided that a suitable 3D template and specific segmentation and classification techniques are used. Compared to other reference methods based mainly on CNNs, our proposal simplifies the 3D reconstruction process by requiring less data and training to obtain a realistic reconstruction of 3D objects.

For future improvements, we plan to address 3D reconstruction from other viewpoints and multiview configurations and to conduct a perceptual study to validate our reconstructions in a virtual environment. In summary, we believe that the work presented in this paper is a step toward automatic and realistic 3D object reconstruction and will be useful in creating 3D content for virtual reality.
